# The relationship between clinical perfectionism and nonsuicidal self‐injury: The roles of experiential avoidance, self‐esteem, and locus of control

**DOI:** 10.1002/jclp.23506

**Published:** 2023-03-04

**Authors:** Thomas Duncan‐Plummer, Penelope Hasking, Kate Tonta, Mark Boyes

**Affiliations:** ^1^ School of Population Health, Faculty of Health Sciences Curtin University Perth Australia; ^2^ Curtin enAble Institute, Faculty of Health Sciences Curtin University Perth Australia; ^3^ Centre for Clinical Interventions Perth Australia

**Keywords:** clinical perfectionism, experiential avoidance, locus of control, nonsuicidal self‐injury, self‐esteem

## Abstract

**Objectives:**

Perfectionism is linked to nonsuicidal self‐injury (NSSI). Individuals with elevated perfectionism tend to avoid undesirable emotions and experience lower self‐esteem, which are associated with NSSI. However, it is unclear if these mechanisms explain the link between clinical perfectionism and NSSI, and if locus of control is involved. We aimed to explore whether experiential avoidance and self‐esteem would mediate the relationship between clinical perfectionism and NSSI, and if locus of control would moderate links between clinical perfectionism and both experiential avoidance and self‐esteem.

**Method:**

As part of a larger study, 514 Australian university students (*M*_age_ = 21.15 years, SD = 2.40; 73.5% female) completed an online survey of NSSI, clinical perfectionism, experiential avoidance, self‐esteem, and locus of control.

**Results:**

Clinical perfectionism was associated with NSSI history, but not with recent NSSI or past year NSSI frequency. Lower self‐esteem, but not experiential avoidance, mediated links between clinical perfectionism and NSSI history, recent NSSI, and NSSI frequency. More external locus of control was associated with NSSI, experiential avoidance, and lower self‐esteem, but locus of control did not moderate pathways between clinical perfectionism and experiential avoidance or self‐esteem.

**Conclusion:**

University students reporting elevated clinical perfectionism may have a tendency to experience lower self‐esteem which is associated with NSSI history, recency, and severity.

## 1 INTRODUCTION

Nonsuicidal self‐injury (NSSI) involves deliberately damaging one's own body tissue without suicidal intent and in a way that is not culturally sanctioned (International Society for the Study of Self‐Injury, 2018). Common self‐injurious behaviors include cutting, burning, and self‐battery, and while distinct, NSSI has been linked to future suicidal behavior (Ribeiro et al., 2016). NSSI primarily functions as a way for individuals to regulate their emotions (Taylor et al., 2018). About 20.2% of university students, compared to 11.5% of the general population, report having engaged in NSSI (Swannell et al., 2014). Although engagement in NSSI usually onsets during adolescence (Plener et al., 2015), there is a second peak onset period at age 20 (Gandhi et al., 2017), with an incidence rate of 10% during the first year of university (Kiekens et al., 2019). Students who engage in NSSI may experience harmful stigmatization (Staniland et al., 2021), reduced social and emotional functioning (Kiekens et al., 2018; Wolff et al., 2019), and lower academic achievement (Kiekens et al., 2016). Collectively, this underscores the need to investigate NSSI among university students. For treatment programs and clinicians to provide better support for students who engage in NSSI, it is crucial to understand and address factors linked to and reasons why students engage in NSSI.

Perfectionism is one factor associated with NSSI (Gyori & Balazs, 2021) and is becoming an increasing concern among university students (Curran & Hill, 2019). There is debate in the literature about how to define perfectionism (see Dunkley et al., 2006; Hewitt et al., 2003; Shafran et al., 2003), though it is typically understood as the tendency to set and strive toward extremely high personal standards, which is often accompanied by self‐criticism and evaluative concerns around meeting these standards (Frost et al., 1990). These perfectionistic self‐critical thoughts and evaluative concerns are linked to psychological distress (Limburg et al., 2017), suicidality (Smith et al., 2018), and burnout (Hill & Curran, 2015). Perfectionism, in particular evaluative concerns over meeting high standards, is considered a transdiagnostic process that likely predicts, maintains, and exacerbates psychopathologies including anxiety, mood, and eating disorders, as well as obsessive‐compulsive disorder (Egan et al., 2011; Limburg et al., 2017). This suggests that perfectionism involving evaluative concerns over meeting high standards may also play a role in maintaining behaviors associated with the experience of distress, such as NSSI.

Shafran et al.'s (2002) model of clinical perfectionism can be used to understand how evaluative concerns over meeting high personal standards may relate to behaviors such as NSSI. Clinical perfectionism involves staking one's self‐worth on meeting high personal standards and continuing to pursue these standards despite negative consequences. Feeling the need to meet high standards to maintain self‐esteem can engender a fear of failure and negative evaluation, which may prompt avoidance of tasks, self‐criticism, and attentional biases toward perceived shortcomings (Shafran et al., 2018). Together, these factors can increase the likelihood that the individual sees their performance as subpar or fails to meet their standards which are continually reappraised as insufficiently demanding (Shafran et al., 2018). Feelings of low self‐worth and self‐criticism associated with being unable to uphold insurmountable standards can lead to distress and the use of harmful coping behaviors to manage this distress (Shafran et al., 2018). Although this provides a basis for understanding how clinical perfectionism and NSSI may be related, research is needed to identify specific mechanisms involved that could be targeted in treatment.

Key components of clinical perfectionism such as self‐criticism and evaluative concerns are associated with NSSI (Gyori & Balazs, 2021). NSSI might function as a way for individuals to manage distress or negative emotions associated with making mistakes, or to exert control over their environment (Hoff & Muehlenkamp, 2009). NSSI could also be used as a means of self‐punishment for individuals with concerns over negative self‐evaluations (Claes et al., 2012). However, few researchers have explored whether clinical perfectionism is directly related to NSSI, or if there are mechanisms through which clinical perfectionism and NSSI are associated that are worth targeting in treatment. Perfectionism may predispose individuals to cascades of rumination and negative affect which are in‐turn associated with NSSI (e.g., Tonta et al., 2022). However, there is also room to consider how other constructs relevant to clinical perfectionism and NSSI such as avoidance (Chapman et al., 2006), self‐esteem (Forrester et al., 2017), and locus of control (Hooley et al., 2010) could explain the relationship between clinical perfectionism and NSSI.

NSSI functions as a way for individuals to regulate their emotions, particularly through facilitating avoidance of undesirable emotions (Taylor et al., 2018). This tendency to avoid undesirable emotional states is referred to as experiential avoidance (Hayes et al., 1996), which has been linked to the maintenance of emotional disorders (Chawla & Ostafin, 2007; Spinhoven et al., 2014) and adverse behaviors such as self‐harm and problematic drug use (Kingston et al., 2010). Experiential avoidance predicts greater frequency of self‐injurious behaviors (Howe‐Martin et al., 2012) and more severe self‐injury (Bentley et al., 2015). According to the Experiential Avoidance Model (Chapman et al., 2006), individuals engage in NSSI to escape from undesirable negative emotions, and this temporary relief from negative affect reinforces the behavior. Using NSSI to alleviate unwanted emotions, although effective in the short term, may counterproductively increase distress and replace alternative emotion regulation strategies, leading to further NSSI engagement in a repetitive cycle (Chapman et al., 2006). The Experiential Avoidance Model has explained links between experiential avoidance and NSSI in undergraduate samples (e.g., Anderson & Crowther, 2012). Students who currently engage in NSSI are more likely to experience more intense emotions and avoid negative cognitions than those without a history of NSSI, or those who previously engaged in NSSI but not in the last year (Anderson & Crowther, 2012).

Self‐critical and evaluative concern aspects of perfectionism are linked to difficulties regulating emotions (Malivoire et al., 2019) and more specifically, experiential avoidance (Moroz & Dunkley, 2015). Individuals with elevated perfectionism who are sensitive to negative evaluations and failure may avoid states where their efforts or self‐presentations are viewed as subpar in the face of unattainably high standards (van der Kaap‐Deeder et al., 2016). Experiential avoidance has also mediated relationships between evaluative concerns perfectionism and both anxiety and depressive symptoms (e.g., Moroz & Dunkley, 2015). Given the overlap in emotion regulation difficulties and experiential avoidance across perfectionism and NSSI, experiential avoidance may mediate the relationship between clinical perfectionism and NSSI, in that NSSI could be linked to avoiding negative emotions arising from setting excessively high standards and self‐evaluative concerns when they are not achieved. Therefore, it may be useful to assess whether experiential avoidance is worth targeting in treatments for perfectionism and NSSI. Although experiential avoidance is one potential mechanism linking clinical perfectionism to NSSI, it is unlikely to predict NSSI entirely (Anderson & Crowther, 2012; Bentley et al., 2015). As such, there may be other variables worth investigating in the relationship between clinical perfectionism and NSSI.

Self‐esteem may be another key factor implicated in the relationship between clinical perfectionism and NSSI. Individuals with lived experiences of NSSI are likely to report lower self‐esteem (Forrester et al., 2017). Lower self‐esteem is also linked to more frequent and severe NSSI (Forrester et al., 2017). Moreover, improving self‐esteem may reduce one's willingness to engage in NSSI (Hooley & St. Germain, 2013). One explanation for the association between lower self‐esteem and NSSI is that individuals may be more likely to engage in NSSI as a form of self‐punishment (see Claes et al., 2012; Nock, 2009) when they have lower regard for themselves (Forrester et al., 2017). Furthermore, individuals with elevated clinical perfectionism may be susceptible to lower self‐esteem through interpreting failure to meet high standards as a negative reflection of their self‐worth (Shafran et al., 2002). Studies in which lower self‐esteem mediates relationships between evaluative concerns perfectionism and depressive symptoms support this link (e.g., Lasota & Kearney, 2017; Moroz & Dunkley, 2015). Students with elevated evaluative concerns are also more likely to base their self‐esteem on achieving high standards, which is in‐turn associated with depressive symptoms (Sturman et al., 2009), and lower self‐esteem (Miegel et al., 2020). As such, it seems plausible that lower self‐esteem associated with negative self‐evaluation in response to perceived failures could link clinical perfectionism to NSSI, perhaps as a means of self‐punishment or regulating unwanted feelings associated with lower self‐esteem.

However, not all individuals with elevated perfectionism avoid undesired emotional states, experience lower self‐esteem, or self‐injure. Perceptions of control and motivation are also important in predicting adaptive or harmful outcomes of perfectionism (Moore et al., 2018; Stoeber et al., 2018), as well as NSSI (Emery et al., 2016). Perceiving one's behaviors as controlled by external demands tends to predict critical, avoidant, and unhealthy outcomes associated with perfectionism, and perceiving one's behaviors as being self‐motivated tends to predict adaptive, reward‐oriented outcomes (Moore et al., 2018; Stoeber et al., 2018). This could have implications for the link between perfectionism and NSSI; if one perceives themselves to be controlled by others, they may be more inclined to adopt avoidance strategies for regulating their emotions (Roth et al., 2019). Conversely, individuals with elevated perfectionism may be less inclined to avoid tasks that may lead to negative emotions if they perceive having control over their actions (Burns et al., 2000). Students who base their self‐worth on meeting demands may be more likely to engage in risky behaviors or experience negative outcomes if they perceive those demands to be controlled by others (e.g., Neighbors et al., 2004).

Considering this, there is scope to see if locus of control increases the likelihood that someone with elevated clinical perfectionism avoids undesirable emotional experiences or experiences lower self‐esteem, which may be associated with NSSI. An external locus of control could plausibly heighten concerns about others' evaluations of the self, predicting avoidance of situations that might lead to negative evaluations. Conversely, an internal locus of control could direct blame inwards for failing to meet one's standards, contributing to lower self‐esteem. However, it may be that an internal locus of control acts as a buffer to low self‐esteem and avoidance in individuals with elevated perfectionism, as the individual may feel that they have a greater control over their ability to maintain their high standards and manage negative self‐evaluation. If locus of control moderates the link between clinical perfectionism and experiential avoidance or self‐esteem, it could provide insights into how perceptions of control may protect against or exacerbate experiential avoidance, low self‐esteem, and potentially NSSI among individuals with elevated clinical perfectionism.

**Figure 1:**
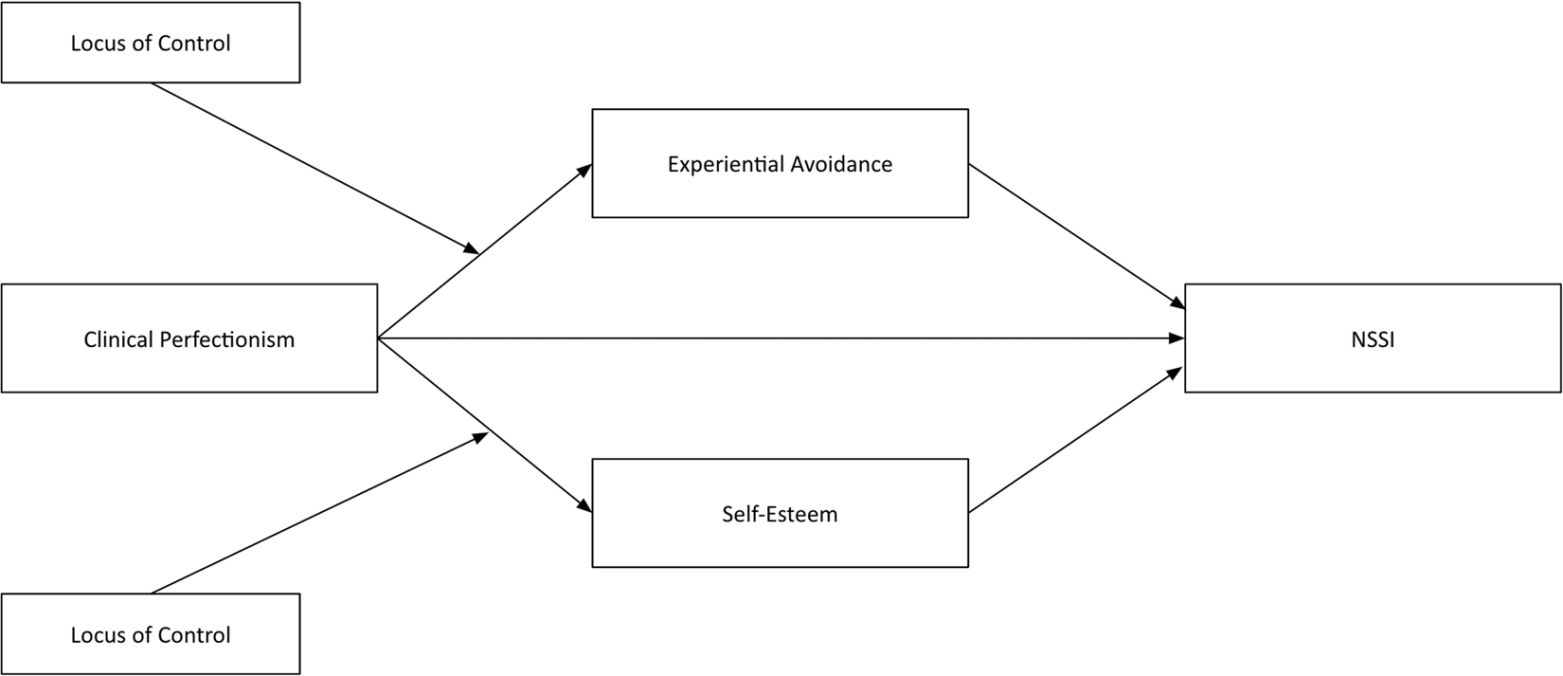
Hypothesized moderated‐mediation model linking clinical perfectionism to NSSI.

The aim of this study was to test the predictions that clinical perfectionism will be associated with history of NSSI along with presence and frequency of NSSI in the last year, that experiential avoidance and self‐esteem will mediate the relationship between clinical perfectionism and NSSI, and that locus of control will moderate the relationships between clinical perfectionism and each mediator (see Figure 1). Specifically, it is hypothesized that clinical perfectionism will be associated with higher levels of experiential avoidance and lower self‐esteem, which will in turn be associated with a history of NSSI, presence of NSSI in the last year, and greater frequency of NSSI. Additionally, we aimed to explore how locus of control may be associated with and moderate the links between clinical perfectionism and both self‐esteem and experiential avoidance.

## 2 METHOD

### 2.1 Participants

The sample comprised 514 Australian university students aged 18–41 years (*M*_age_ = 21.15, SD = 2.40, 73.5% identified as female). Most participants (97.3%) were undergraduate students. About 40.7% of participants (*n* = 209) who completed the survey reported having engaged in NSSI. The 192 participants who reported the frequency of their self‐injury over the past year (*M*_age_ = 21.38, SD = 2.00, 86.5% identified as female) comprised the sample for the NSSI recency and frequency analyses.

### 2.2 Measures

#### 2.2.1 NSSI

Section 1 of the Inventory of Statements About Self Injury (ISAS; Klonsky & Glenn, 2009) was used to assess the type and frequency of participants' NSSI. Participants were shown the ISSS (2018) definition of NSSI and were asked, “Have you ever engaged in non‐suicidal self‐injury?”, to which they responded no (coded as 0) or yes (coded as 1). Participants who reported NSSI estimated the age at which they first self‐injured, the frequency of their self‐injury over the last year on a six‐point scale (1 = *none* to 6 = *five or more times*), and their primary method of NSSI. The ISAS has adequate test‐retest reliability over 4 weeks (*r* = 0.85) and 1 year (*r* = 0.68; Glenn & Klonsky, 2011).

#### 2.2.2 Clinical perfectionism

The Clinical Perfectionism Questionnaire (CPQ; Fairburn et al., 2003) contains 12 items (e.g., “Have you felt a failure as a person because you have not succeeded in meeting your goals?”), each measuring a participant's level of clinical perfectionism on a four‐point scale (1 = *not at all* to 4 = *all of the time*). Participants rate how well the statement described them over the past month. The two reverse coded items were not analysed in keeping with Shu et al. (2020) recommendations. The remaining 10 items were computed into a global score as per Howell et al. (2020), with higher scores indicating a higher level of clinical perfectionism. The CPQ has predicted stress, along with symptoms of depression and anxiety in nonclinical samples (Chang & Sanna, 2012), and had sound internal consistency (*α* = 0.78) and validity in a community sample of adults (Howell et al., 2020). The CPQ had good test–retest reliability over 4 months in an undergraduate student sample (Dickie et al., 2012). In this study, internal consistency was good (*α* = 0.81).

#### 2.2.3 Experiential avoidance

The Brief Experiential Avoidance Questionnaire (BEAQ; Gámez et al., 2014) is a condensed version of the Multidimensional Experiential Avoidance Questionnaire (MEAQ; Gámez et al., 2011). The BEAQ contains 15 items (e.g., “I am quick to leave any situation that makes me feel uneasy”), each measuring participants' tendency to engage in experiential avoidance on a six‐point scale (1 = *not at all* to 6 = *all of the time*). The measure covers experiential avoidance in several forms, for instance, through task avoidance or suppressing emotions. A higher total score indicates a greater level of experiential avoidance. Gámez et al. (2014) reported good internal consistency for the BEAQ in student samples (*α* = 0.86) and strong correlations with the well‐validated and reliable MEAQ along with several avoidance measures. In this study, internal consistency was excellent (*α* = 0.90).

#### 2.2.4 Self‐esteem

The Rosenberg Self‐Esteem Scale (RSE; Rosenberg, 1965) contains 10 items (e.g., “On the whole, I am satisfied with myself”), each measuring a participant's perceived self‐worth on a four‐point scale (1 = *strongly agree* to 4 = *strongly disagree*). A higher total score shows a more positive perception of oneself. The RSE has good internal consistency in undergraduate student samples (*α* = 0.90) and strongly correlates with other self‐esteem measures (Robins et al., 2001). In this study, internal consistency was excellent (*α* = 0.91).

#### 2.2.5 Locus of control

The Locus of Control of Behavior Scale (LCBS; Craig et al., 1984) contains 17 items (e.g., “My life is controlled by outside actions and events”), each measuring participants' beliefs about whether their behavior is controlled internally or externally on a six‐point scale (0 = *strongly disagree* to 5 = *strongly agree*). A higher total score indicates more perceived external control over behaviors, and a lower total score indicates more perceived internal control over behaviors. Craig et al. (1984) reported good validity and test–retest reliability over one week (*r* = 0.90) and six months (*r* = 0.73), along with good internal consistency (*α* = 0.79) with a sample of 100 university students. In this study, internal consistency of the LCBS was adequate (*α* = 0.78).

### 2.3 Procedure

The Human Research Ethics Committee at Curtin University approved the recruitment and data collection procedures. As part of a larger study, Australian university students (specifically those with and without a history of NSSI) were invited to complete an online survey via university recruitment systems and Facebook advertisements from September 2018 to September 2019. We advertised NSSI as a focus of the study. Students were awarded course credit for participation or went into a draw to win one of ten $25 gift cards or an iPad. Information about the study was communicated to students via an online information sheet before them choosing to give consent. The survey took 45–60 min to complete. After completing the survey, participants were given information about how to access mental health resources and counseling support should they wish to.

### 2.4 Data analysis

SPSS v28.0 was used to clean data, and to check assumptions of linear and logistic regression given linear relationships between scale variables, the binary outcomes of both NSSI history and NSSI in the last year, and NSSI frequency as an ordinal outcome. Intercorrelations and descriptive statistics were computed for variables of interest and used to screen for potential confounders (e.g., age and gender). We tested the proposed moderated‐mediation models in MPlus v8.2 using maximum likelihood estimation (ML) and the logit link function for categorical outcomes. Estimates and 95% confidence intervals for direct and indirect effects were generated using 10,000 bootstrap samples. We standardized scale variables to limit multicollinearity (Tabachnick & Fidell, 2013) and used unstandardized coefficients and 95% confidence intervals to interpret effects in the models.

## 3 RESULTS

### 3.1 Data cleaning and screening

Missing value analysis showed that less than 2% of data was missing for variables assessed at the item level, which was missing completely at random, *χ*² (2354) = 2395.17, *p* = 0.27. As such, missing values were imputed using expectation maximization (Tabachnick & Fidell, 2013). No univariate outliers were detected. Two cases were identified as multivariate outliers and were removed from the analysis due to a lack of variability in the response patterns. Assumptions of linear regression were all met. Regarding assumptions of logistic regression, there was no evidence of multicollinearity, and predictor variables were normally distributed. However, the logit linearity assumption was not met for self‐esteem. Although this may not directly affect the model's fit, it increases the risk of making Type II errors (Tabachnick & Fidell, 2013). As such, the significance and magnitude of the relationship between self‐esteem and NSSI in this sample may be underestimated.

### 3.2 Preliminary analysis

Of the participants who reported lived experience of NSSI, 117 (56%) reported having self‐injured within the last year, 75 (35.9%) reported no self‐injury in the past year, and 17 (8.1%) did not disclose the recency or frequency of their self‐injury. Approximately 48.7% of the participants who self‐injured in the past year self‐injured five or more times during this period. Age of NSSI onset ranged from 4 to 23 years (*M* = 13.74, SD = 3.01). Cutting was the most frequently reported primary method of NSSI (49.5%), followed by banging or hitting oneself (12.8%), and scratching (12.2%).

As seen in Table 1, variables correlated in the expected directions. Participants who reported NSSI also reported higher levels of clinical perfectionism and experiential avoidance, lower self‐esteem, and more external locus of control compared to those without NSSI history. Participants with higher levels of clinical perfectionism and experiential avoidance, lower self‐esteem, and more external locus of control were more likely to have self‐injured in the last year, and reported more frequent self‐injury during this period. Female participants were more likely to report a history of NSSI than males, *χ²* (1, *N* = 514) = 25.65, *p* < 0.001, *Ѵ* = 0.22 (47.0% vs. 21.7%). As such, gender was statistically controlled in the NSSI history model.

**Table 1:** Intercorrelations and descriptives for final sample (*N* = 514).

	No NSSI (*n* = 305)	NSSI (*n* = 209)	NSSI 12 (*n* = 117)						
Variable	*M* (SD)	*M* (SD)	*M* (SD)	1	2	3	4	5	6
1. Age	21.04 (2.63)	21.34 (2.03)	21.32 (1.97)	‐					
2. Gender^a^	‐	‐	‐	−0.07	‐				
3. Clinical perfectionism	23.25 (5.11)	25.84 (5.75)	26.68 (5.72)	0.03	0.19***	‐			
4. Experiential avoidance	46.38 (13.24)	52.32 (13.49)	54.84 (13.50)	−0.12**	0.18***	0.23***	‐		
5. Self‐esteem	28.55 (5.19)	23.04 (6.25)	21.59 (6.01)	0.003	−0.18***	−0.29***	−0.51***	‐	
6. Locus of control	49.21 (8.96)	54.43 (9.21)	55.62 (9.16)	−0.03	0.14***	0.24***	0.45***	−0.56***	‐
NSSI^a^	‐	‐	‐	0.06	0.22***	0.23***	0.21***	−0.43***	0.27***
NSSI 12 months^a^	‐	‐	‐	−0.04	0.09	0.15*	0.25***	−0.29***	0.16*
NSSI frequency	‐	‐	4.54 (1.62)	−0.03	0.09	0.15*	0.20**	−0.30***	0.19*

Abbreviations: Gender coded—1, male; 2, female. NSSI coded—0, no NSSI history; 1, NSSI history. NSSI 12 Months coded: 0, no NSSI in the last 12 months; 1, NSSI in the last 12 months. NSSI, nonsuicidal self‐injury; NSSI 12, NSSI in the last 12 months.

^a^ Point biserial correlation.

* *p* < 0.05 (two‐tailed)

** *p* < 0.01 (two‐tailed)

*** *p* < 0.001 (two‐tailed).

### 3.3 Model testing

**Figure 2:**
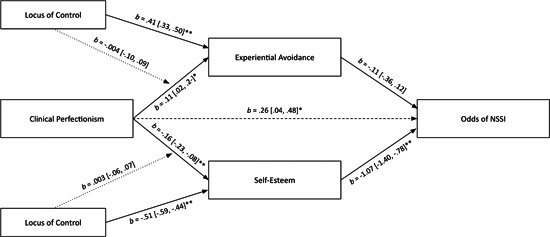
Moderated‐mediation model linking clinical perfectionism to odds of NSSI. Unstandardized coefficients and 95% confidence intervals are reported. Coefficients with odds of NSSI as the outcome are in logit scale. Dashed lines are nonsignificant paths. *p < 0.05 (two‐tailed) and **p < 0.001 (two‐tailed).

**Figure 3:**
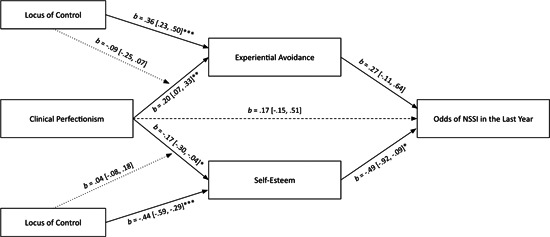
Moderated‐mediation model linking clinical perfectionism to odds of NSSI in the last year. Unstandardized coefficients and 95% confidence intervals are reported. Coefficients with odds of NSSI in the last year as the outcome are in logit scale. Dashed lines are nonsignificant paths. *p < 0.05 (two‐tailed) **p < 0.01 (two‐tailed) ***p < 0.001 (two‐tailed).

**Figure 4:**
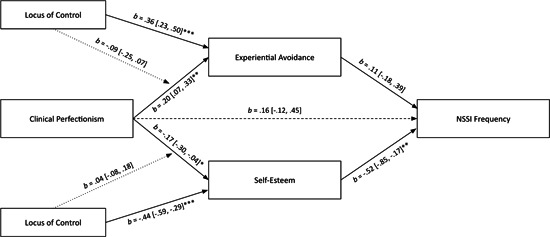
Moderated‐mediation model linking clinical perfectionism to NSSI frequency. Unstandardized coefficients and 95% confidence intervals are reported. Coefficients with NSSI Frequency (ordinal) as the outcome are in logit scale. Dashed lines are nonsignificant paths. *p < 0.05 (two‐tailed), **p < 0.01 (two‐tailed), and ***p < 0.001 (two‐tailed).

In the models, clinical perfectionism was directly associated with NSSI history (Figure 1), but not with NSSI in the last year (Figure 2) or NSSI frequency (Figure 3). Clinical perfectionism was significantly associated with experiential avoidance and lower self‐esteem. Experiential avoidance was not significantly associated with any of the NSSI measures. Lower self‐esteem, however, was significantly associated with NSSI history, NSSI in the last year, and NSSI frequency. Experiential avoidance did not significantly mediate links between clinical perfectionism and NSSI history (*b* = −0.01, 95% CI [−0.06, 0.01]), NSSI in the last year (*b* = 0.05, 95% CI [−0.01, 0.17]), or NSSI frequency (*b* = 0.02, 95% CI [−0.03, 0.10]). Conversely, lower self‐esteem did mediate the links between clinical perfectionism and NSSI history (*b* = 0.17, 95% CI [0.08, 0.27]), NSSI in the last year (*b* = 0.08, 95% CI [0.01, 0.23]), and NSSI frequency (*b* = 0.09, 95% CI [0.01, 0.21]). More external locus of control was significantly associated with experiential avoidance and lower self‐esteem, but it did not significantly moderate the pathways between clinical perfectionism and experiential avoidance or self‐esteem (Figure 4).

## 4 DISCUSSION

The purpose of this study was to understand why clinical perfectionism may be related to NSSI, focusing on the mediating roles of experiential avoidance and self‐esteem. We also aimed to explore whether locus of control would moderate the links between clinical perfectionism and both experiential avoidance and self‐esteem. Overall, clinical perfectionism was directly associated with NSSI history, but not with the recency or frequency of NSSI. Self‐esteem, but not experiential avoidance, mediated links between clinical perfectionism and NSSI history, NSSI in the last year, and frequency. Locus of control did not moderate associations between clinical perfectionism and experiential avoidance or self‐esteem. These results help to clarify that evaluating oneself on the basis of meeting unrealistically high personal standards is associated with lower self‐esteem, and it is primarily lower self‐esteem which explains the recency, severity, and higher likelihood of NSSI in this context. These findings also have implications for understanding how experiential avoidance and locus of control play a role in the link between clinical perfectionism and NSSI.

Students who reported elevated clinical perfectionism were more likely to have a history of NSSI, which is consistent with research showing that individuals with evaluative concerns around meeting high standards are more likely to have self‐injured at some point in their lifetime (Gyori & Balazs, 2021). However, given that clinical perfectionism was not directly related to recent NSSI or NSSI frequency, clinical perfectionism may be less useful for explaining the severity of NSSI among students who currently self‐injure. It may be more informative to focus on the finding that students with lower self‐esteem were more likely to have self‐injured in the last year and self‐injured more frequently, which aligns with research showing that lower self‐esteem is linked to greater NSSI severity (Forrester et al., 2017). Together, this suggests that although basing self‐esteem on meeting unattainably high standards coincides with NSSI history, it may be more clinically useful to focus on the experience of low self‐esteem for understanding the recency and severity of NSSI.

If students base their self‐esteem on the process of meeting unrealistically high personal standards, they may also be more inclined to negatively evaluate themselves, which is in‐turn associated with NSSI. This may capture the premise that basing self‐esteem on the achievement of extremely high personal standards is a key component of why clinical perfectionism and evaluative concerns are linked to harmful behaviors (Egan et al., 2011; Shafran et al., 2002). Basing self‐esteem on achievement means that students may be vulnerable to lower self‐esteem through interpreting failure to meet high standards as a negative reflection of themselves (Shafran et al., 2002). In‐turn, students with lower self‐esteem may engage in NSSI as a means of managing aversive emotions related to self‐critical thoughts and feelings of low self‐worth (Forrester et al., 2017), or because lower self‐esteem can facilitate engagement in NSSI as a form of self‐punishment (Hooley & St. Germain, 2013). However, the cross‐sectional nature of this study means it is not possible to draw temporal links between students' clinical perfectionism, self‐esteem, and NSSI. Engagement in NSSI could also elicit feelings of shame or low self‐esteem given that NSSI is often stigmatized (Staniland et al., 2021). Additionally, individuals may seek to achieve high standards to regulate existing feelings of low self‐worth (Malivoire et al., 2019). Considering this, longitudinal research is needed to elucidate temporal links between clinical perfectionism, self‐esteem, and NSSI.

Experiential avoidance was associated with NSSI in the univariate analyses, which is consistent with research showing that individuals who tend to avoid undesirable emotions are more likely to engage in NSSI (Angelakis & Gooding, 2021). Experiential avoidance was more strongly related to NSSI in the past year than NSSI history, which may suggest that experiential avoidance is particularly relevant for understanding recent rather than past engagement in NSSI (see Anderson & Crowther, 2012). However, experiential avoidance was not significantly associated with NSSI in the models. Therefore, a tendency to avoid undesirable emotions may be less useful for understanding why clinical perfectionism is linked to NSSI after accounting for self‐esteem and locus of control. This may reflect shared variance across emotion‐related constructs in predicting NSSI (see Haywood et al., 2022). Given that individuals with elevated clinical perfectionism may primarily avoid emotions or situations linked to negative self‐evaluations (Shafran et al., 2002), perhaps experiential avoidance is only relevant to NSSI in this context when it encompasses feelings or regulation of lower self‐esteem. In keeping with Shafran et al.'s (2002) premise that negative self‐evaluation around failure to achieve high standards is the main component tying clinical perfectionism to harmful behaviors, it may be worth focussing on students’ self‐worth rather than their avoidance of undesirable emotions in general when addressing the link between clinical perfectionism and NSSI.

However, experiential avoidance can also be distinct from low self‐esteem among individuals with elevated perfectionism (Moroz & Dunkley, 2015), and only certain forms of experiential avoidance such as procrastination and thought suppression may be relevant to NSSI in university students (see Bentley et al., 2015; Howe‐Martin et al., 2012). Therefore, assessing a student's general tendency to avoid unwanted affect may not fully capture the relationship between experiential avoidance and NSSI in this context. It may be worth seeing if tests of the current model using a dimensional approach to experiential avoidance (see Gámez et al., 2011) provide a more nuanced picture of how clinical perfectionism, experiential avoidance, and NSSI are related.

Students reporting elevated clinical perfectionism, experiential avoidance, lower self‐esteem, and NSSI perceived their behaviors to be controlled mainly by external sources. This is consistent with more external locus of control being associated with heightened evaluative concerns (Periasamy & Ashby, 2002) and engagement in NSSI (Hooley et al., 2010). These findings may fit with the idea that clinical perfectionism and NSSI operate as ways for individuals to exert control over their circumstances where they generally perceive a lack of personal control over behaviors and outcomes (Hoff & Muehlenkamp, 2009; Shafran et al., 2002). The results also support the idea that students who believe their behaviors and outcomes in life are controlled externally are more likely to engage in avoidance strategies for regulating their emotions (Roth et al., 2019) and may experience lower self‐esteem (Hooley et al., 2010), which may have negative implications for NSSI.

However, the extent to which students with higher clinical perfectionism reported experiential avoidance or lower self‐esteem was not dependent on their perceived control over behaviors. This is unusual considering that perceiving oneself to be controlled by external demands is associated with negative correlates of perfectionism (Stoeber et al., 2018). One explanation is that general perceptions of control over behavior are less relevant than the underlying motivations, attributions, or value associated with meeting high standards in specific domains (see Stoeber et al., 2018). Furthermore, students may experience internal, external, or a combination of both loci of control in specific domains (Rotter, 1975), and only certain domains may be relevant to clinical perfectionism and NSSI. For example, although a student may generally perceive their behavior to be controlled externally, when it comes to meeting high standards of academic work, they may feel they are entirely responsible for their achievement. As such, one's general locus of control may not capture nuances in locus of control for students with elevated clinical perfectionism. Considering this, it may be worth seeing if individuals have a heightened internal or external locus of control in specific domains related to their high standards which may have implications for their self‐esteem and NSSI. Longitudinal research is also needed to see if an external locus of control tends to precede staking self‐esteem on high standards and engagement in NSSI, or if external locus of control acts as a way for students to rationalize existing feelings of low self‐worth in the face of high expectations.

### 4.1 Theoretical and clinical implications

Given that clinical perfectionism is associated with other harmful behaviors such as disordered eating, finding that clinical perfectionism also plays a role in NSSI supports the idea that clinical perfectionism may be a transdiagnostic process (Egan et al., 2011). This research also supports Shafran et al.'s (2002) clinical perfectionism model by demonstrating that basing self‐worth on achieving high personal standards is associated with NSSI. It is therefore important to consider the context surrounding students' self‐esteem, such as the belief that high standards must be met to maintain it, in addressing the link between perfectionism and NSSI. Although clinical perfectionism may be helpful for understanding why students have self‐injured at some point in their lifetime, lower self‐esteem appears to be more relevant for understanding current presentations of NSSI. It may also be useful to account for other variables related to why individuals avoid undesirable emotions in the relationship between experiential avoidance and NSSI. A general propensity to avoid undesired emotions may be less relevant for understanding whether students engage in NSSI when other constructs such as self‐esteem are considered. As the Experiential Avoidance Model focuses on the maintenance of repetitive self‐injury (Chapman et al., 2006), it may be useful to assess specific types and functions of experiential avoidance in relation to current NSSI, rather than as a trait to discern between those who have or have not engaged in NSSI. This research also suggests that an external locus of control is linked to increased frequency and engagement in NSSI, but that it may be useful to conceptualize locus of control as a domain‐specific construct (see Rotter, 1975) when investigating its role in the link between clinical perfectionism and NSSI.

Clinicians could help students appraise their self‐worth in ways that do not rely on meeting extreme standards, as per Shafran et al. (2018), to potentially manage negative self‐evaluations which are in‐turn associated with NSSI. Online programs targeting maintaining factors of clinical perfectionism, such as the overdependence of self‐esteem on meeting high standards, can reduce levels of clinical perfectionism (e.g., Shafran et al., 2017) and improve self‐esteem (e.g., Kothari et al., 2019). It may be worth assessing if these programs are also capable of targeting NSSI via reductions in clinical perfectionism and reappraisals of self‐worth among university students.

### 4.2 Limitations and suggestions for future research

The current study's correlational design means that causal links cannot be drawn between clinical perfectionism and NSSI. The cross‐sectional nature of the data also means that the temporal links between and context surrounding clinical perfectionism and NSSI are unclear. In future efforts to understand if individuals with elevated clinical perfectionism use NSSI to regulate feelings of low self‐worth or as a form of self‐punishment, it would be useful to incorporate functions of NSSI into the model. It would also be useful to understand how specific avoidance and emotion regulation strategies, fluctuations in self‐esteem, and domain‐specific loci of control are associated with clinical perfectionism and NSSI using methods that are more ecologically valid, such as ecological momentary assessment (see Rodríguez‐Blanco et al., 2018). The indirect effect of clinical perfectionism on NSSI operating through self‐esteem was small, especially for recent engagement in NSSI. Considering this, other factors such as psychological distress, emotion regulation difficulties (see Malivoire et al., 2019), and rumination (e.g., Tonta et al., 2022) should also be taken into account to explain the link between clinical perfectionism and proximal NSSI. Although we specifically aimed to investigate clinical perfectionism and NSSI in university students, advertising NSSI as a focus of the study may have coincided with the higher incidence of NSSI in this sample relative to other undergraduate samples (cf. Swannell et al., 2014), meaning that findings from the NSSI history model may not generalize to wider populations. It could be useful to recruit a heterogenous group of university students and individuals from general or clinical populations to further investigate the relationship between clinical perfectionism and NSSI.

## 5 CONCLUSION

Researchers and clinicians should acknowledge that staking self‐esteem on achieving unrealistically high personal standards, and lower self‐esteem associated with this, is linked to a higher likelihood of university students engaging in NSSI. In light of this, students’ general tendency for experiential avoidance and their overall locus of control, although still important, may be less useful for understanding why elevated clinical perfectionism is linked to NSSI. With this in mind, researchers should investigate temporal links between clinical perfectionism, self‐esteem, and NSSI, along with specific forms of experiential avoidance and domain‐specific locus of control, to better explain how clinical perfectionism and NSSI may be related and treated.

## CONFLICT OF INTEREST STATEMENT

The authors declare no conflict of interest.

### PEER REVIEW

The peer review history for this article is available at https://www.webofscience.com/api/gateway/wos/peer-review/10.1002/jclp.23506.

## Data Availability

The data that support the findings of this study are available from the corresponding author upon reasonable request.
